# Supplementary figures and images for: External validation of the PAR-Risk Score to assess potentially avoidable hospital readmission risk in internal medicine patients
Source: PLoS One. 2021 Nov 23;16(11):e0259864. doi: 10.1371/journal.pone.0259864 (PMC8610256; doi:10.1371/journal.pone.0259864)

## S1 Figure. Frequency of predictors of PAR vs. non-PAR patients.


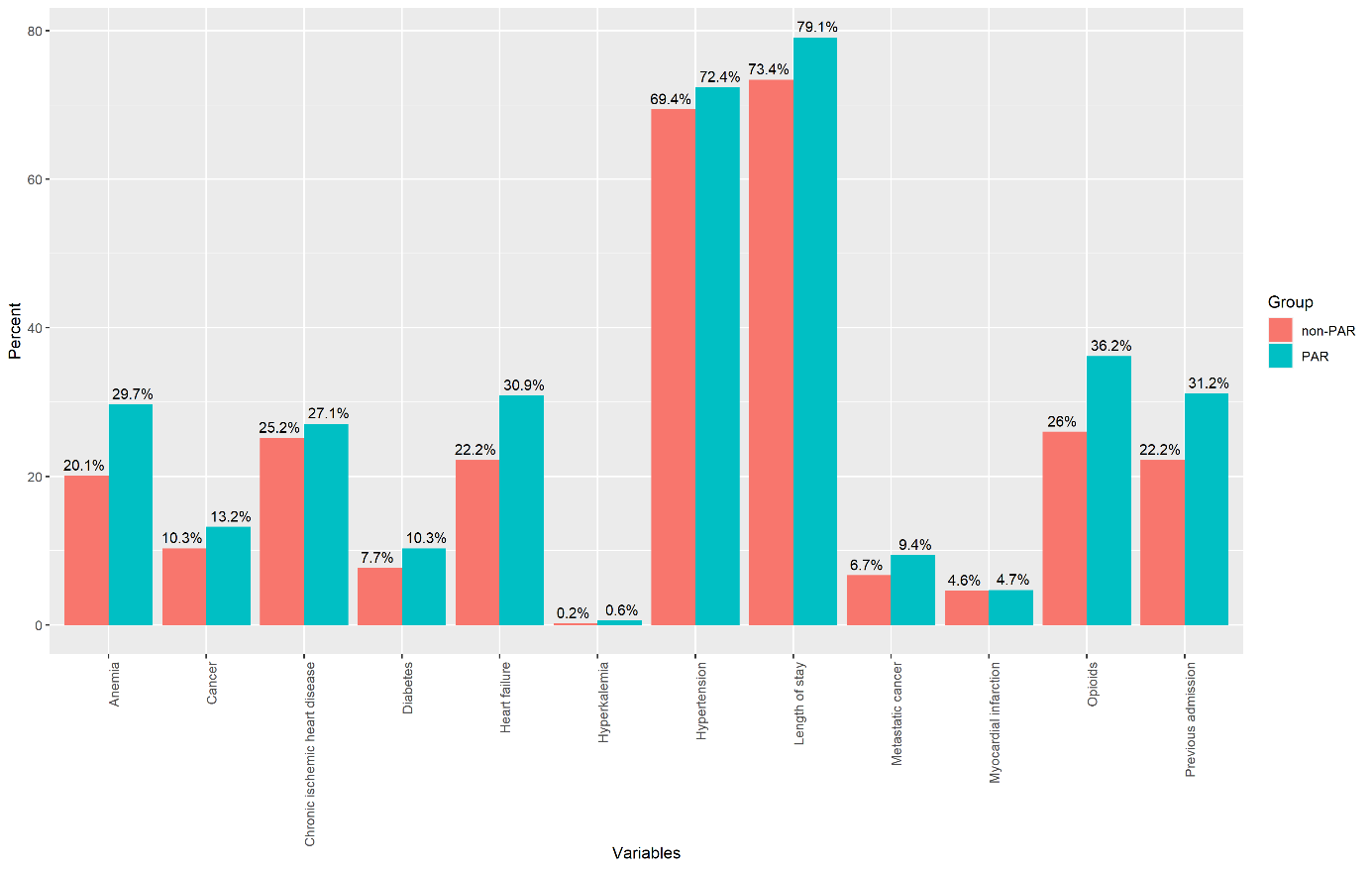

Supplement: S1 Fig — (DOCX) [file pone.0259864.s001.docx]

## S3 Figure. Receiver operating curve of the univariable logistic regression.

C-statistic = 0.605.


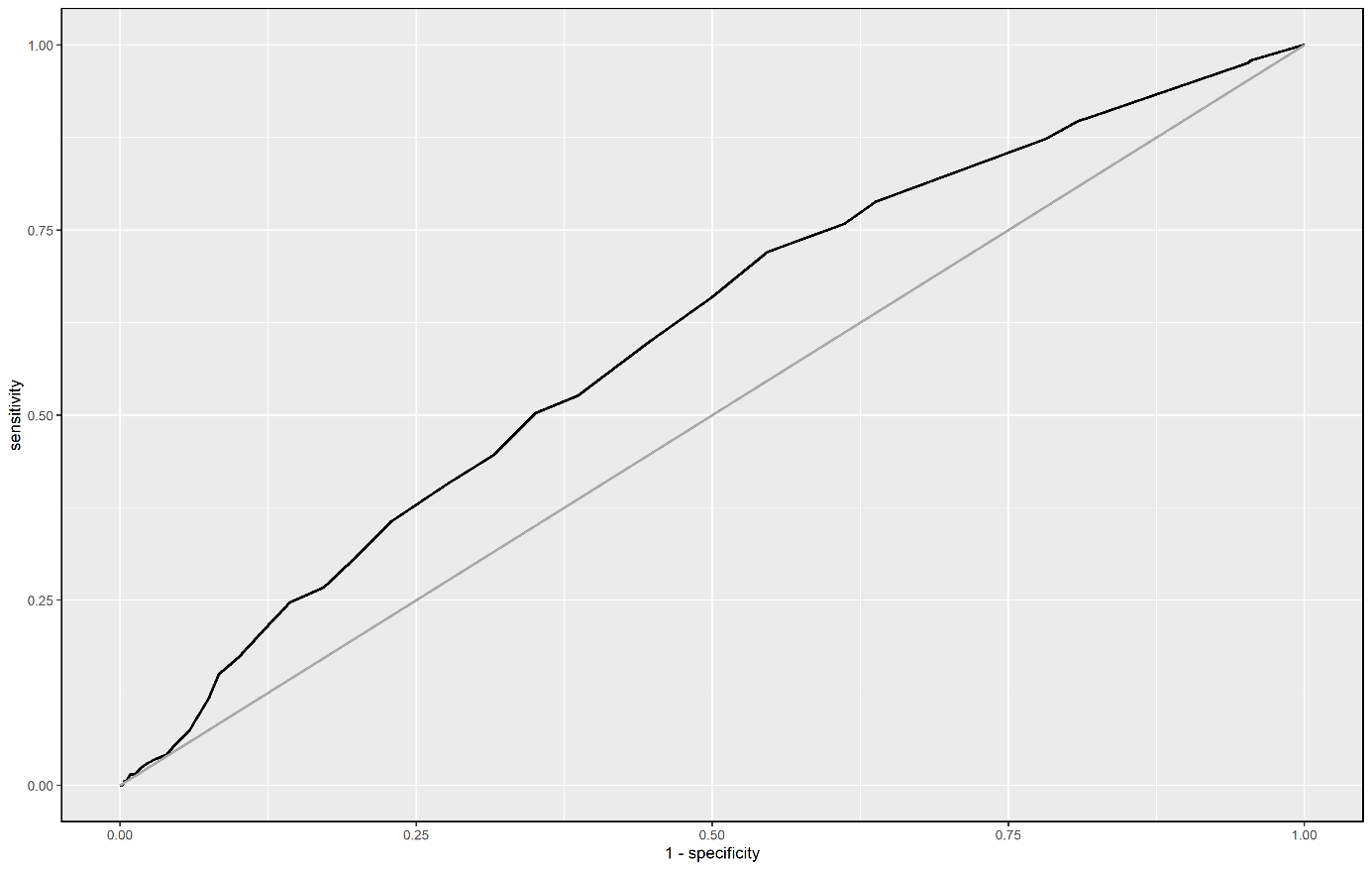

Supplement: S3 Fig — C-statistic = 0.605. (DOCX) [file pone.0259864.s003.docx]
