# Supplementary material for: External validation of the PAR-Risk Score to assess potentially avoidable hospital readmission risk in internal medicine patients
Source: PLoS One. 2021 Nov 23;16(11):e0259864. doi: 10.1371/journal.pone.0259864 (PMC8610256; doi:10.1371/journal.pone.0259864)

## S2 Figure. Comparison of the distribution of raw PAR-Risk Score values in non-PAR and PAR group.

The dashed line indicates the original threshold levels (<3, 3-10, >10). The dot-dashed line indicates the adapted threshold levels (<12, 12-25, >25).


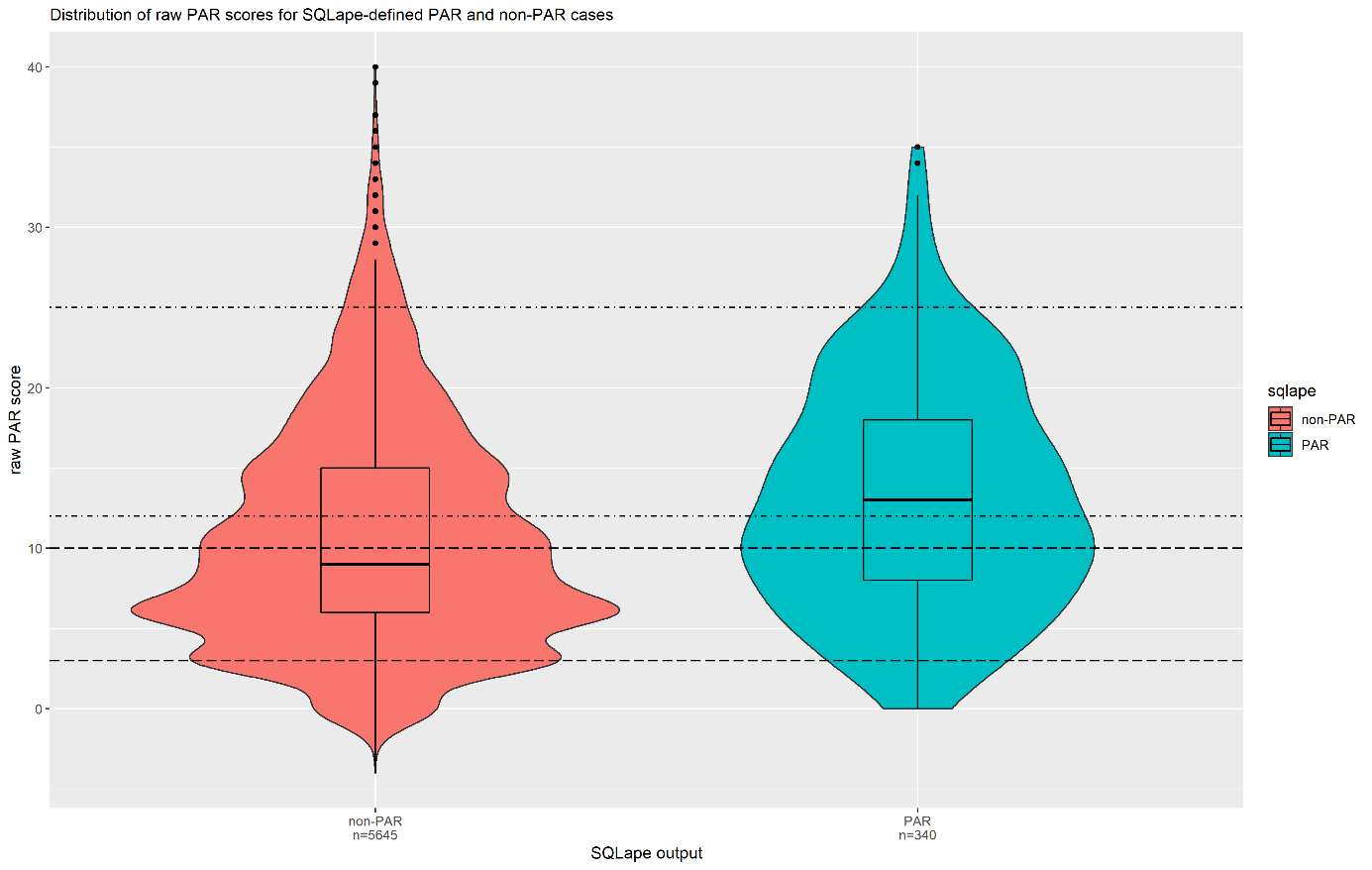

Supplement: S2 Fig — The dashed line indicates the original threshold levels (<3, 3–10, >10). The dot-dashed line indicates the adapted threshold levels (<12, 12–25, >25). (DOCX) [file pone.0259864.s002.docx]
