# Supplementary material for: External validation of the PAR-Risk Score to assess potentially avoidable hospital readmission risk in internal medicine patients
Source: PLoS One. 2021 Nov 23;16(11):e0259864. doi: 10.1371/journal.pone.0259864 (PMC8610256; doi:10.1371/journal.pone.0259864)
Supplement: S1 Table — (DOCX) [file pone.0259864.s004.docx]

## S1 Table. Predictors and points for the calculation of the raw PAR-Risk Score.

| **Predictor** | **Points** |
| --- | --- |
| **Administrative characteristics** | |
| Admission in previous 6 months | 8 |
| Length of hospital stay | 3 |
| **Comorbidities** | |
| Anaemia | 2 |
| Heart failure | 4 |
| Hypertension | 3 |
| Acute myocardial infarction | -4 |
| Chronic ischemic heart disease | 5 |
| Diabetes with organ damage | 9 |
| Cancer | 4 |
| Metastatic carcinoma | 6 |
| **Medications** | |
| Opioids | 3 |
| **Lab results** | |
| Hyperkalaemia (serum potassium level > 5.5 mmol / L) | 4 |

Adapted from Blanc A-L, Fumeaux T, Stirnemann J, Lozeron ED, Ourhamoune A, Desmeules J, et al. Development of a predictive score for potentially avoidable hospital readmissions for general internal medicine patients. PLOS ONE. 2019;14: e0219348. doi:10.1371/journal.pone.0219348
