# Supplementary material for: External validation of the PAR-Risk Score to assess potentially avoidable hospital readmission risk in internal medicine patients
Source: PLoS One. 2021 Nov 23;16(11):e0259864. doi: 10.1371/journal.pone.0259864 (PMC8610256; doi:10.1371/journal.pone.0259864)
Supplement: S2 Table — (DOCX) [file pone.0259864.s005.docx]

## S2 Table. Results of the univariable logistic regression using the raw PAR-Risk Score values to predict PAR by SQLape.

| **Univariable Logistic Regression** | Estimate | 95% Confidence interval | Standard error | P value |
| --- | --- | --- | --- | --- |
| Raw PAR-Risk Score | 0.04 | 0.03, 0.06 | 0.01 | p < .001 |
