# Supplementary material for: External validation of the PAR-Risk Score to assess potentially avoidable hospital readmission risk in internal medicine patients
Source: PLoS One. 2021 Nov 23;16(11):e0259864. doi: 10.1371/journal.pone.0259864 (PMC8610256; doi:10.1371/journal.pone.0259864)
Supplement: S3 Table — (DOCX) [file pone.0259864.s006.docx]

## S3 Table. Goodness of fit test statistic of the univariable logistic regression.

| **Le Cessie - van Houwelingen - Copas – Hosmer unweighted sum of squares test**  **for global goodness of fit** | | | | |
| --- | --- | --- | --- | --- |
| Observed value* | Expected value \| H0 | Standard Deviation | Z-value | P-value |
| 318.968 | 318.567 | 0.144 | 2.763 | 0.006 |

*Notes: *Sum of squared errors*
