# Supplementary material for: External validation of the PAR-Risk Score to assess potentially avoidable hospital readmission risk in internal medicine patients
Source: PLoS One. 2021 Nov 23;16(11):e0259864. doi: 10.1371/journal.pone.0259864 (PMC8610256; doi:10.1371/journal.pone.0259864)
Supplement: S4 Table — The predicted risk was calculated by applying the scoring of the original study to each patient and then calculating the mean predicted risk by group. (DOCX) [file pone.0259864.s007.docx]

## S4 Table. Coefficients of the multivariable regression of the original study.

The predicted risk was calculated by applying the scoring of the original study to each patient and then calculating the mean predicted risk by group.

| **Predictor** | **Regression coefficients** |
| --- | --- |
| Intercept | -3.39 |
| Admission in previous 6 months | 0.83 |
| Length of hospital stay | 0.27 |
| Anaemia | 0.22 |
| Heart failure | 0.27 |
| Hypertension | 0.29 |
| Acute myocardial infarction | -0.45 |
| Chronic ischemic heart disease | 0.53 |
| Diabetes with organ damage | 0.81 |
| Cancer | 0.34 |
| Metastatic carcinoma | 0.68 |
| Opioids | 0.29 |
| Hyperkalaemia (serum potassium level > 5.5 mmol / L) | 0.30 |
