# Supplementary material for: External validation of the PAR-Risk Score to assess potentially avoidable hospital readmission risk in internal medicine patients
Source: PLoS One. 2021 Nov 23;16(11):e0259864. doi: 10.1371/journal.pone.0259864 (PMC8610256; doi:10.1371/journal.pone.0259864)
Supplement: Attachment — Submitted filename: R1_Answers_PAR_PLOS.docx [file pone.0259864.s009.docx]

Revision of Manuscript on the external validation of the PAR-Risk Score

Answers to Reviewers’ Comments

The authors would like to send their gratitude to the reviewers and the editor for their positive and constructive feedback. The comments and shared thoughts improved the overall work and added important details to the article.

To ease correspondence, we copied all comments into a table below. The reviewers’ comments are on the left side; the authors’ answers are on the right side. Indicated pages and lines refer to the clean version of the revised manuscript (track changes off).

We applied the following color scheme to our answers: Green states implementation, yellow states implementation which may be regarded as only partial, and orange states currently no implementation into the main body/only further clarification.

# Reviewer #1

| **Comment for the author** |  | **Authors response to the reviewer** |
| --- | --- | --- |
| Wouldn't it have been an option to validate AND update the model to fit better with the new population? There has been an adaptation in the risk score cut offs but what about new variables that show better association and discrimination for a risk score? |  | We agree with the reviewer that this would have been an option. However, based on the results and learnings from this external validation, we now would have approached the prediction of PAR differently for this dataset. For this reason, we chose to focus on extensively characterising the performance of the existing model. We showed the changes in performance by adapting the cut-off values or comparing different performance indicators for multi-class problems. |
| As I understood cantonal is a Swiss healthcare system but might be difficult to understand for those that are not Swiss. Perhaps the authors could use secondary care as it is more widely understood? |  | We apologize for the confusion and thank the reviewer for highlighting it. The word canton describes the official member states (“cantons”) of the Swiss confederation and “cantonal” is the corresponding adjective, often approximating the size of the hospitals.  Thus, we **changed** “cantonal hospital” to “**tertiary teaching** hospital” for the international readers throughout the manuscript. |
| The authors state that the derivation cohort was a primary care hospital but it is unclear to me what this means. Could the authors elaborate on this? |  | We thank the reviewer for notifying us of this potentially confusing wording. The level of care determines the services provided by the hospital, the higher the level the more specialised services are provided. To be consistent, we **re-phrased** the hospital level matching the original publication:  “In 2019, Blanc et al. [14] published the internally validated Potentially Avoidable Readmissions Risk Score (PAR-Risk Score), developed with a dataset of **one tertiary university teaching hospital and one regional hospital**” [page 5, lines 86-87]  And on page 6, lines 96-97:  «This cohort study used data from hospitalisations of a 360-bed **tertiary teaching hospital** in Baden, Switzerland [15] between December 2016 and November 2018.” |
| Why did the authors specify the age as above 65 years? 40% of the patients in the derivation cohort were under 65 years old? This was not an exclusion criteria in the initial study and could have potentially introduced bias and an underestimation of the c-statistic. |  | Our dataset only consisted of patients aged 65 years and older as described on page 6, lines 99-101. This was not a specification by us, but rather a limitation in the available data.  We **re-phrased** the following sentence within the limitations section to state this more clearly [page 18, lines 306-308]:  “Our external validation was conducted in older patients than in the development study, and had a required hospitalisation stay longer than 48 hours. **This was a limitation of the available data** and could have potentially influenced the model’s performance.” |

| How much missing data was there? In the manuscript it is written that population mean was imputed, however this is an unrealiable form of imputing missing data as it shrinks standard errors and could alter the associations between variables. I would suggest to use multiple imputation or a Bayesian methods for single imputation. |  | There were 432 (7.2%) patients with missing data on serum potassium levels and 94 (1.5%) patients with missing data on dispensed drugs. This is reported in the results section on page 9, lines 174-177: “Due to missing data, we set the PAR-Risk Score variable hyperkalaemia to 0 for 432 patients (7.2%). The 94 (1.5%) patients who were excluded due to missing data on dispensed drugs showed an underrepresentation of PAR cases with just one PAR case.”  We agree with the reviewer that applying a more sophisticated imputation technique would have been preferred and we thank the reviewer for the suggestions. However, as this was an external validation, we aimed to reproduce the original study as closely as possible, thus, potentially having the same methodological limitations.  Upon thorough discussions within our group, we concluded not to use any form of imputation in addition to using “the last observation carried forward” method: we are convinced that the missing potassium serum levels were not missing at random. From a clinical perspective, patients with suspected deviations from normal serum potassium levels would not have been discharged without measuring it. Missing potassium lab values may, hence, be a hint towards normal potassium levels. Thus, we think it is appropriate to set their PAR risk score hyperkalaemia variable to 0.  We **re-phrased** the following sentence to clarify this [page 7, lines 126-128]:  “Within individual patients, the most recent serum potassium levels were carried forward. **When there was no value available at all, we assumed that those were not missing at random (i.e., the patient had normal serum potassium levels)**.”  And at page 9, lines 174-175:  «Due to missing data, **we set the PAR-Risk Score variable hyperkalaemia to 0 for 432 patients (7.2%).**”  To assess the impact of our approach, we now **performed an additional sensitivity analysis** by setting the hyperkalaemia variable for all patients with missing values to 1. This is described on page 7, lines 128-129:  «A sensitivity analysis was performed by setting the hyperkalaemia variable for all patients with missing values to 1.»  With results described on page 13, lines 209-210:  «The sensitivity analysis on patients with missing serum potassium levels showed only small changes in the C statistic: 0.600 (95% CI: 0.570-0.633) by setting hyperkalaemia to one.» |
| --- | --- | --- |
| How did the authors choose the new cut offs in the adapted risk score? |  | The new cut-offs were chosen the same way as it was done in the development phase. We recalculated the tertiles based on the raw PAR-Risk Score value of our study participants.  This is described in the method section on page 8, lines 151-153:  “This grouping was redone with adapted threshold levels, which were re-calculated by grouping the patients into tertiles based on the raw PAR-Risk Score values of our study participants, analogously to the development phase.” |

# Reviewer #2

| **Comment for the author** |  | **Answer to the editor** |
| --- | --- | --- |
| Title a. I suggest adding “potentially avoidable” to the outcome “hospital readmission risk” to clearly represent the outcome that was measured. |  | We agree with the reviewer and thank for the suggestion.  We changed the title to:  “External validation of the PAR-Risk Score to assess **potentially avoidable** hospital readmission risk in internal medicine patients” |
| Introduction a. The medical context is well-explained but should be shortened. |  | We agree and shortened the introduction. Word count before: 602; now: 473 (79%). The introduction now reads:  “Potentially avoidable readmissions (PAR) are unforeseen readmissions related to a previously known affliction occurring within a specified time interval [1]. PAR-rates vary between 5 to 79% and are increasingly being used as benchmarks for quality of care, hospital outcomes, and cost reduction measures [2,3]. For Switzerland, the rates vary between 3.8% to 5.6% and generally depend on the level of care the hospital provides [4]. Of the many reasons for hospital readmissions investigated, adverse drug events have been shown to account for 13% of 30-day readmissions to an academic hospital in the US. Of these, 93% were classified as preventable and 49% were caused by inappropriate prescribing [5][6]. Interventions to reduce readmission rates have been explored by focussing on improved discharge planning and reducing adverse drug events, including patient education, telephone follow-up, home visits, and transition coaching [5]. However, no multicomponent intervention program has yet brought consistent evidence to sufficiently reduce readmission rates [5]. Furthermore, they are reported to be time consuming and expensive [7].  To address these issues, readmission prediction models have been developed and validated [7]. These models stratify patients according to their risk of readmission using readily available electronic health care information to calculate a risk score early in the hospitalisation in order to target interventions [8,9]. A recent systematic review on prediction models by Mahmoudi et al. identified 41 studies reporting on prediction models. Of these, 17 predict the risk of readmission on all inpatients while the rest of the models focus on a specific patient group [7]. Only eight studies reported sensitivity and specificity, implementation in the electronic medical record system seemed rare, and no model had been externally validated [10].  In Switzerland, the national Striving for Quality Level and Analyzing of Patient Expenses (SQLape) software [11] is being used to identify PAR within 30 days after discharge. The underlying screening algorithm identifies unplanned readmissions to the same hospital that are related to the initial diagnosis and occur within 30 days of hospital discharge with a specificity and sensitivity of 96% [1]. However, the screening algorithm of SQLape only works in retrospect and, hence, cannot be used for targeted preventive interventions. In 2019, Blanc et al. [12] published the internally validated Potentially Avoidable Readmissions Risk Score (PAR-Risk Score), developed with a dataset of one tertiary university teaching hospital and one regional hospital. The PAR-Risk Score is a 12-items prediction model, where the weights of the regression coefficients were transposed to a simpler scoring system. The internal validation showed a C-statistic of 0.688 (95% CI 0.655 to 0.72), which is close to the models reviewed by Mahmoudi et al. [7] and the already externally validated Swiss HOSPITAL score [13,14].  In this study, we aimed to provide the first external validation of the PAR-Risk Score using data from an older internal medicine patient cohort of a Swiss tertiary teaching hospital.” |
| Methods a. The authors used correctly the TRIPOD check-list; |  | We thank the reviewer for acknowledging our efforts. |
| b. I suggest replacing “population” by “sample” or “participants”; |  | We thank the reviewer for the suggestion. Throughout the manuscript, we **replaced** the word “population” with “participants” or “patients” depending on the context. |
| c. To consider an external validation the patients may be different from the derivation cohort. The main differences between the author’s sample and the development population should be emphasised explaining the reasons for that to constitute a sample for external validation. Also, the shared points: internal medicine patients; |  | We thank the reviewer for this important comment. For this reason, we added the patient characteristic of the derivation cohort to our table 1. Thus, the main differences in the two patient cohorts can be seen when looking at the patient characteristics. We, additionally, **added** the following sentences on page 6, lines 96-101:  “This cohort study used data from hospitalisations of a 360-bed tertiary teaching hospital in Baden, Switzerland [15] between December 2016 and November 2018. As in the development study, we focused on internal medicine patients. The data were derived from routinely collected electronic health records (EHR). The study included patients hospitalised for at least 48 hours and aged 65 years and older, as selected for another study [16]. These dataset characteristics had not been applied to the patient sample in the development study.” |
| d. The inclusion criteria (age > 65 and hospitalizations longer than 48 hours) were not clearly explained; |  | These criteria were given as we used a dataset that was initially built to address another clinical research question, see page 6, lines 99-101: “The study included patients hospitalised for at least 48 hours and aged 65 years and older, as selected for another study [16]. These dataset characteristics had not been applied to the patient sample in the development study.»  We hope that we clarified the approach with the changes made because of the comment above. As we see having these criteria as a limitation of our external limitation, we addressed it in the limitations section. |
| e. The age criteria could move the purpose of this external validation not only for the validation in another Centre within the same country where the PAR-Risk Score was derived (geographic validation, same country, other region), but also in a specific subgroup of patients, the elderly – these items should be incorporated at aims; |  | We thank the reviewer for highlighting this specification. We are also convinced that a rehospitalisation model should be tested in an older population, which is at inherent risk for rehospitalisation, and see it as a rationale for doing an external validation in our given dataset. We **re-phrased** the sentence on page 5, line 92-93 as follows:  “In this study, we aimed to provide the first external validation of the PAR-Risk Score using data from an **older** internal medicine patient cohort of a Swiss tertiary teaching hospital.” |
| f. What were the main reasons for having missing values? Why did the authors use different approaches to deal with missing data in serum potassium and medication? Please clarify these topics. |  | We answered the reviewer’s comment about serum potassium just below [comment (g)].  Missingness in medication, on the other hand, is a clinically unlikely scenario for patients hospitalised longer than 48 hours and older than 65 years of age. We were, hence, not able to find justifications for any assumptions, and chose to exclude these 94 cases (1.7%) from further analyses. |
| g. A single imputation using sample mean was used to serum potassium levels in 514 (8.6%) patients. As the score uses a cut-off value of 5.5 mmol/L it is probably that all of those patients did not enter with a value which count to scoring 4 points, please discuss. |  | We discussed our decisions for using our approach on missing potassium levels in our response to reviewer #1.  We now **performed an additional sensitivity analysis** by setting the hyperkalaemia variable for all patients with missing values to 1. This is described on page 7, lines 128-129:  «A sensitivity analysis was performed by setting the hyperkalaemia variable for all patients with missing values to 1.»  With results described on page 13, lines 209-210:  «The sensitivity analysis on patients with missing serum potassium levels showed only small changes in the C statistic: 0.600 (95% CI: 0.570-0.633) by setting hyperkalaemia to one.»  We now included explanations on why we chose not to use sophisticated imputation techniques in the limitations section, which read:  “Based on clinical considerations, we decided against using imputation techniques for missing potassium values and information on dispensed drugs. If irregularities of potassium levels were to be expected, appropriate lab work would have been ordered. To investigate the impact of our considerations, we performed a sensitivity analysis, only showing small changes in the C statistic. No documented medication dispensing is an unlikely scenario for hospitalised patients over 65 years of age, suggesting incorrect data entry. We, hence, decided on excluding these 94 patients.” [page 18, lines 310-317]  We would like to thank the reviewer for additionally pointing us towards an unfortunate error, for which we apologise: the true number of missing serum potassium values was 432 (7.2%), not 514 (8.6%). This error was introduced by the overlap of patients with missing medication data and potassium values. |
| h. The authors performed missing imputation regarding medication and some sensitivity analyses considering two types of imputation and complete-cases analysis – this information is distributed between missing data and statistical analyses. Please report the type of missing imputation that was done within missing data subsection; |  | We agree with the reviewer and removed the following sentence form the statistical analysis subsection:  “A sensitivity analysis for the exclusion of patients with missing content on dispensed medication was performed by setting the opioid variable for all these patients to either 0 or 1.”  We **added** the following sentence to the missing data subsection [page 7, lines 131-133]:  “The sensitivity analysis on the 94 excluded patients was performed by setting the opioid variable for all these patients to either 0 or 1.” |
| i. Please clarify lines 205-206, “the sensitivity analysis on the 94 excluded patients” or “the sensitivity analysis with/adding the 94 excluded patients”? |  | We thank the reviewer for the clarification request and reworded on page 7, line 132:  “The sensitivity analysis **on the 94 excluded patients** was performed by setting the opioid variable for all these patients to either 0 or 1.” |
| j. I’m not sure if any model updating was done or only the creation of new risk subgroups according to PAR-score distribution (please verify and complete 10e from TRIPOD accordingly). |  | We thank the reviewer for noting this inconsistency in the reporting guideline. Besides PAR-Risk Score redistribution, we did not perform any model updating. TRIPOD Item 10e was now **changed** to **N/A**. |
| 4. Results a. Please report how many PAR cases were reported on the 94 patients excluded due to missing data on dispensed drugs; |  | We agree with the reviewer that this should be reported. There was only **one** PAR case in the 94 missing patients. We added this to page 9, lines 175-177:  “The 94 (1.5%) patients who were excluded due to missing data on dispensed drugs showed an underrepresentation of PAR cases with just one PAR case.” |
| b. Please be cautious when reporting that 5.7% (PAR in the external cohort) is comparable with 7.5% (PAR in the derivation cohort) without any quantitative measure for comparison; |  | We thank the reviewer for highlighting the impact of our linguistic choices. We agree that using “comparable” would necessitate a quantitative measure, especially in the results section. We re-phrased the sentence as follows:  “Of the eligible patients, 340 patients (5.7%) were identified as having experienced a PAR by the SQLape software, whereas it was 562 (7.7%) in the derivation patient cohort.” [page 9, lines 169-171]  We, additionally, needed to specify 7.7% for the derivation cohort, because 7.5% referred to the derivation + internal validation of the development study. We apologize for this unfortunate transcription error. |
| c. Table 1 – Please add the % of SQLape defined PAR cases in the derivation cohort; |  | We thank the reviewer for noting this inconsistency.  Table 1 now shows SQLape cases in the derivation cohort of 562 (7.7%) |
| d. Table 2 and Table 3 – Please add N by groups Non-PAR and PAR; |  | We thankfully added the group sizes to both tables (Table 2 and Table 3). |
| e. Figure 2 – Please note that the total exclusions (n=2267) do not correspond to the sum of the 4 exclusion criteria; |  | We sincerely thank the reviewer for noting this. We now added the correct numbers to the flow chart (Figure 2). |
| f. Figure S3 C-statistic (0.602) does not correspond to the C-statistic reported in the text (0.605) – line 202; |  | We thank the reviewer for noting this unfortunate mismatch, for which we apologize. Changed the Figure S3 legend to C-statistic = 0.605. |
| g. Table S3 – add observed value; |  | We thank the reviewer for this comment, which we addressed by writing “observed value” and denoting below the table that this corresponds to the sum of squared errors. |
| h. Regarding the risk stratification, medium and high-risk groups were at higher odds of having a PAR than low risk group considering the adapted threshold; while using the original threshold, only the high-risk group was significantly at a higher odd of having PAR versus low-risk category. Please reformulate lines 210-212; |  | We thank the reviewer for mentioning this specification. We **re-phrased** as follows [page 13, lines 215-218]:  “When comparing medium and high risk groups to low risk groups, patients were at higher odds of being hospitalised **for the adapted threshold levels. For the original threshold levels only the high risk group was significantly at higher odds of being hospitalised.**” |
| i. Consider adding a calibration plot to table 5 to simplify reading and interpretation. |  | We thank the reviewer for this valuable suggestion and agree that a calibration plot would improve understanding. We now **added** a weighted calibration plot as figure 3. [page 13, line 208].  We consequentially **re-phrased** the following sentences in the methods section [page 7, lines 141-143]  “Additionally, we visualised the calibration by plotting the observed proportion at risk per PAR Risk Score point versus the predicted risk for each point weighted by the respective number of participants.”  And in the results section [page 12, line 205-206]  “The calibration plot indicated a lack of fit (Fig. 3), which was also supported by the goodness-of-fit test with a *p*-value of 0.01.” |
| 5. Limitations a. The development study was not conducted in an older patient sample (~40% of patients were <65 years old) – please verify line 300; |  | We thank the reviewer for highlighting this linguistic error that led to a misunderstanding. We **re-phrased** the sentence as follows [page 18, line 306-307]:  “Our external validation was conducted in older participants **than** in the development study, and had a required hospitalisation stay longer than 48 hours.” |
| b. Please consider including the limitations of the missing imputations methods. Would a multiple imputation provide more consistent results? |  | We now included explanations on why we chose not to use sophisticated imputation techniques in the limitations section, which read:  “Based on clinical considerations, we decided against using imputation techniques for missing potassium values and information on dispensed drugs. If irregularities of potassium levels were to be expected, appropriate lab work would have been ordered. To investigate the impact of our considerations, we performed a sensitivity analysis, only showing small changes in the C statistic. No documented medication dispensing is an unlikely scenario for hospitalised patients over 65 years of age, suggesting incorrect data entry. We, hence, decided on excluding these 94 patients.” [page 18, lines 310-316] |
| For PAR outcome and internal medicine patients’ population there are diverse prediction models. Therefore, an external validation of those prediction models on the same cohort can provide a comparison of the predictive performance between those models. Indeed, it is surprising that the HOSPITAL score reached similar discriminatory power and overall accuracy in the original/derivation cohort of the PAR-Risk score. It would be expectable that the new score to be superior since it was optimally designed to fit that data/sample. It would be interesting to check how the HOSPITAL score performs in this external cohort, as the medication seems to be the main difference between the PAR-Risk Score and HOSPITAL score and the missing information on medication data is one of the drawbacks of this external validation. |  | Whilst we agree that comparing the PAR-Risk Score head-to-head to the HOSPITAL score would have been interesting, the comparison has already been made for the original publication of the PAR-Risk Score. Additionally, our dataset does not provide haemoglobin values that are necessary for the full potential of the HOSPITAL score. Given these circumstances, we hope that the reviewer accepts our decision to solely focus on evaluating the external validity of the PAR-Risk Score. |

# Journal Requirements

| **Comment for the author** |  | **Authors response to the reviewer** |
| --- | --- | --- |
| Please ensure that your manuscript meets PLOS ONE's style requirements, including those for file naming. |  | We apologize for not catching the requirements on file names. We now renamed the figure files and separated the supplementary data into individual files with their respective file names. |
| In ethics statement in the manuscript and in the online submission form, please provide additional information about the patient records/samples used in your retrospective study. Specifically, please ensure that you have discussed whether all data/samples were fully anonymized before you accessed them and/or whether the IRB or ethics committee waived the requirement for informed consent. If patients provided informed written consent to have data/samples from their medical records used in research, please include this information. |  | We gladly added information on the ethics approval. The new section reads: “The Swiss ethics committee approved the protocol for the study for which the data were originally extracted (EKNZ Project ID: 2018-01000). The committee also approved the amendment for the study presented here. The data were extracted anonymously, informed patient consent was not required.” [page 8, lines 161-165] |
| PLOS ONE has specific requirements for studies that are presenting a new method or tool as the primary focus, including a newly developed or modified questionnaire or scale (https://journals.plos.org/plosone/s/submission-guidelines#loc-methods-software-databases-and-tools.) One requirement is that the questionnaire or scale must be openly available under a license no more restrictive than CC BY. In light of this, before we proceed, please include a copy of your questionnaire or scale as a Supporting Information file (in the original language) or provide a link if it is available through an online repository. |  | The original model, which we externally validated, was also published in PLOS ONE under the CC BY license. We cited this original publication throughout the manuscript and additionally provided the scoring (PAR Risk Score) itself in the supplement (S1 Table). |
| We note that the grant information you provided in the ‘Funding Information’ and ‘Financial Disclosure’ sections do not match. |  | We apologize for the mismatch, which we corrected now. |
| Please include your full ethics statement in the ‘Methods’ section of your manuscript file. In your statement, please include the full name of the IRB or ethics committee who approved or waived your study, as well as whether or not you obtained informed written or verbal consent. If consent was waived for your study, please include this information in your statement as well. |  | We gladly added information on the ethics approval. The new section reads: “The Swiss ethics committee approved the protocol for the study for which the data were originally extracted (EKNZ Project ID: 2018-01000). The committee also approved the amendment for the study presented here. The data were extracted anonymously, informed patient consent was not required.” [page 8, lines 161-165] |
